# Is sympathetic nervous system involved in the etiopathogenesis of idiopathic scoliosis? - A preliminary animal study in bipedal C57BL/6J mice model

**DOI:** 10.1186/1748-7161-10-S1-O12

**Published:** 2015-01-19

**Authors:** Zhen Liu, Jing Guo, Tao Wu, Bangping Qian, Zezhang Zhu, Feng Zhu, Jian Jiang, Yong Qiu

**Affiliations:** 1 Spine Surgery, the Affiliated Drum Tower Hospital of Nanjing University Medical School, Nanjing, China

## Summary

A preliminary animal study to investigate whether the incidence of scoliosis or the magnitude of curves can be changed by pharmacological sympathectomy in bipedal C57BL/6J mice model.

## Introduction

The sympathetic nervous system (SNS), as an important part of autonomic nervous system, through its hypothalamic neuroendocrine control of puberty, menarche and skeletal growth may contribute significantly to the pathogenesis of adolescent idiopathic scoliosis (AIS). Hyperactivity of the SNS has been reported to result in lower body mass index (BMI), lower bone mineral density (BMD), and longer extremities, which are commonly seen in AIS patients. The objective of this study was to investigate the effect of sympathectomy by way of pharmacological agents on the development of scoliosis in bipedal mice model.

## Methods

Sixty female 3-week-old C57BL/6J mice were divided into three groups after amputations of forelimbs and tails. Group 1 received daily intraperitoneal injection of 0.9% saline (5 ml/kg/day); while Group 2 and 3 received sympathectomy by daily intraperitoneal injection of propranolol (20 mg/kg/day) and guanethidine sulfate (40 mg/kg/day), respectively. Posteroanterior X-rays were obtained at 20th week. Curves were measured using Cobb method and scoliosis was defined as a Cobb angle of > 10°. Incidence of scoliosis and severity of curves were compared among groups.

## Results

There were 17 (85%) mice presented scoliosis in Group 1; whereas 11 (55%) and 10 (50%) mice presented scoliosis in Group 2 and 3, respectively. The incidence of scoliosis was found to be higher in Group 1, but the statistical significance was just marginal (P=0.046). As for curve magnitudes, the mean Cobb angle was 20.5°±9.2° in Group 1, 10.2°±6.8° in Group 2, and 11.7°±7.9° in Group 3. The mean Cobb angle of Group 1 was significantly greater than those of Group 2 and 3 (P<0.01), but there was no significant difference in mean Cobb angle between Group 2 and 3.

## Conclusion

Sympathetic nervous system may be involved in the development and progression of scoliosis in bipedal C57BL/6J mice model. Sympathectomy do not seem to dramatically decrease the incidence of scoliosis, probably due to that bipedalism itself may also be a cause of scoliosis in this animal model.

